# Chicago Medical Society

**Published:** 1874-06-01

**Authors:** Will. T. Montgomery


### CHICAGO MEDICAL SOCIETY.

### REGULAR SEMI-MONTHLY MEETING MAY 18, 1874.

*Reported by Will. T. Montgomery.*

THE Society met in the parlor of the Gault House, President Dr. Quine in the chair. Special order of business for the meeting: Lecture by Prof. Delafontaine on the Spectroscopic Appearance of Blood.

The President referred to the continued absence of a portion of the Committee on Membership, and suggested that some action be taken in reference to it. Dr. Earle moved that a temporary committee be appointed to act in the absence of the regular one. Carried. The President appointed as such committee Drs. Paoli, Ingals and Strong. The Society next listened to the Lecture announced for the evening.

In his Lecture, the Professor referred to the controversy between different investigators as to whether or not it is possible to distinguish, by means of the Spectroscope, between fresh and old blood. He, with Dr. C. P. Simon, had instituted experiments for the purpose of determining this, and had used four specimens of blood as follows: First, cattle blood two years old. Second, his own blood four weeks old. Third, oxen blood three days old. Fourth, blood two days old from a fatal case of hydrophobia. The fresh specimens present two dark bands in the spectrum; one in the green and one in the yellow. The old specimens present an additional dark band in the red. This is very distinct in the specimens two years old, but in the specimens four weeks old it is merely a faint line. The additional band is invariably present in old blood. The Professor thinks that the spectroscope is more reliable as a means of determining the qualities of blood than the microscope, and predicts for it a wide range of usefulness in examinations of the urine for bile, &c. He referred to some peculiarities presented by the blood of the hydrophobia patient. It remained fluid much longer than healthy blood, but decomposition began earlier. Two days after the death of the patient, bacteria began to appear in the blood and multiplied very rapidly. Two varieties of fungus were also discovered. The Lecturer and Dr. Simon exhibited two specimens of blood by means of the spectroscope which members of the Society were invited to examine.

A vote of thanks was given the Professor for his interesting lecture.

Dr. Worrell, President of the Illinois State Medical Society, having come in during the progress of the meeting, was introduced by the President, Dr. Quine, and addressed to the Society a few happy and well chosen remarks.

After miscellaneous business was disposed of a motion to adjourn prevailed.

